# Forecast of the incidence, prevalence and burden of end-stage renal disease in Nanjing, China to the Year 2025

**DOI:** 10.1186/s12882-016-0269-8

**Published:** 2016-06-13

**Authors:** Ling Sun, Lu-Xi Zou, Yu-Chen Han, Han-Ming Huang, Zhao-Ming Tan, Min Gao, Kun-Ling Ma, Hong Liu, Bi-Cheng Liu

**Affiliations:** Department of Nephrology, the Affiliated Xuzhou Hospital of Medical College of Southeast University, No.199, Jiefang South Road, Xuzhou, 221009 Jiangsu China; Department of Information Management, the Affiliated Xuzhou Hospital of Medical College of Southeast University, No.199, Jiefang South Road, Xuzhou, 221009 Jiangsu China; Institute of Nephrology, Zhongda Hospital, Southeast University, No. 87, Ding Jia Qiao Road, Nanjing, 210009 Jiangsu China; Nanjing Municipal Human Resources and Social Security Bureau, Nanjing, China

**Keywords:** End stage renal disease, Forecast, Incidence, Prevalence, Medical insurance, Health care costs

## Abstract

**Background:**

There are limited data on the trends of incidence or prevalence of end stage renal disease (ESRD) in China. To assist in future planning for the ESRD program, the trends of incidence, prevalence and health care costs were analyzed and forecasted to the year 2025 by modeling of historical data from 2004 through 2014.

**Methods:**

Nanjing urban employee basic medical insurance (NJUEBMI) data were obtained from the Nanjing Medical Insurance Information System from 2004 to 2014. The time series forecasting system in SAS 9.4 was used. Each variable was independently forecasted by the fittest model, which was selected automatically or manually.

**Results:**

The forecasting models demonstrated mean percent errors of −2.49 to 5.62 %, relative to the observed values. The R-square values for the forecasting models ranged from 0.756 to 0.997. On the basis of trends in the historical data, the models projected that the average annual increase in the NJUEBMI population was 4.77 %, with forecasted values of 5,029,270 in 2025 (95 % CI, 4,960,423-5,098,117). The incidence and prevalence of ESRD were projected to increase by 1.19 and 1.95 % annually and were expected to reach 250.5 pmp (95 % CI, 247.7–253.3) and 1505 pmp(95 % CI, 1450–1560) by 2025. Additionally, the costs associated with ESRD were forecasted to increase at a growth rate of 5.80 % for healthcare costs and 7.25‰ for per capita medical expenses, with forecasted values of ¥600.3 million ($92.4 million) (95 % CI, 541.8–658.9) and ¥99.0 thousand ($15.2 thousand) (95 % CI, 98.6–99.3), respectively, by 2025. The incidence and prevalence of kidney transplantation were projected to decrease by 6.58 and 9.79 % annually.

**Conclusions:**

These projections suggest that the incidence, prevalence, healthcare costs, and per capita medical expenses of ESRD would increase in the NJUEBMI population. They provide a basis for discussing the trends of ESRD in China and facing the challenges from the ESRD program.

**Electronic supplementary material:**

The online version of this article (doi:10.1186/s12882-016-0269-8) contains supplementary material, which is available to authorized users.

## Background

Chronic kidney disease (CKD) has become a worldwide public health problem, with increasing incidence, prevalence, poor prognoses and high health care costs. End stage renal disease (ESRD) has caused heavy economic burdens to patients and governments, particularly in developed countries [1].

Many developed countries and regions have established a national registry system to up-date information on ESRD, evaluate renal replacement therapy (RRT) services and develop future healthcare policy, like the USA (USRDS), the United Kingdom (UK Renal Registry), Europe (ERA-EDTA Registry) and Taiwan (TSN Dialysis Registry). Although China has developed a national web-based renal registry system since 2010 (the Chinese Renal Data System, CNRDS), high quality data are still lacking due to the incomplete registry [2].

Based on dialysis facility-level data, in 2010, the incidence and prevalence of hemodialysis (HD) were 147.3 per million population (pmp) and 509 pmp in Beijing [3], and the incidence and prevalence of dialysis were 114.8 pmp and 774 pmp in Shanghai [4]. The incidence and prevalence in both cities were much lower than in Taiwan, Japan and the USA [5, 6]. However, the mobility of ESRD patients is extremely high in Beijing and Shanghai, and the data might not reflect the true burden of ESRD.

Nanjing, the capital city of Jiangsu Province, is a relatively developed city with a stable population. The health insurance program of the Nanjing government has offered reimbursement for the costs of RRT, including HD, peritoneal dialysis (PD) and kidney transplantation since 2000, which was much earlier than most cities in China. In Nanjing, the following types of basic medical insurance are provided by the government: urban employee basic medical insurance (UEBMI), urban resident basic medical insurance (URBMI), and rural cooperative medical insurance (RCMI). Of which, the reimbursement proportion of UEBMI is the highest. The patients covered by Nanjing UEBMI (NJUEBMI) could afford RRT and would not withdraw from RRT for economic reasons. In other words, the treatment gap between needed and actual RRT is the smallest in ESRD patients of NJUEBMI population. Furthermore, the population migration in Nanjing is much less than in Beijing and Shanghai. Thus, the data from the population covered by NJUEBMI might reflect the true status of the incidence and prevalence of ESRD in urban populations in the developed areas of China.

To better understand the changing trend of ESRD in Nanjing, we analyzed the data from the NJUEBMI population to forecast the trends of incidence, prevalence and health care costs of ESRD patient population in the next decade. Our results should be very important to the government in facing the challenges from increasing burden of ESRD.

## Methods

### Data sources and collection

The Nanjing Medical Insurance Information System (NJMIIS) was established in 2000, and all the Medical Insurance Information data were recorded, including all the NJUEBMI population. The information on patient hospital visits was submitted to the NJMIIS automatically, including the diagnosis, medicine prescribed, and health care costs.

The data on the size of NJUEBMI population and the number of treated ESRD patients, including new and long-term ESRD, dialysis and kidney transplantation patients, were obtained from the NJMIIS, on December 31 of each year, from 2004 to 2014. The NJUEBMI covered only 16 % of whole Nanjing population in 2004 and increased to 36 % in 2014 (Additional file 1: Table S1). This study was approved by the ethics committee of Zhongda Hospital affiliated to Southeast University.

### Definition of ESRD, incidence and prevalence

ESRD was defined as having irreversible damage in renal function due to a state of uremia and requiring maintenance RRT. The RRT modalities included HD, PD and kidney transplant. The annual incidence, calculated as the number of new patients entering ESRD between 1 January and 31 December divided by mid-year NJUEBMI population counts, was expressed as per million population (pmp). The prevalence, calculated as the number of ESRD patients on December 31 divided by the same point NJUEBMI population counts, was also expressed as pmp.

### Data processing and statistical analyses

The SAS (version 9.4) “forecast” procedure, which includes a time series forecasting system, was used to forecast the variables for each patient group to 2025 [7]. Each variable was independently forecasted by “Fit Model Automatically” to achieve the fittest model. If the automatically chosen model could not fit the data or minimize the residuals well, we manually searched for the model with best goodness-of-fit statistical values, according to the data distribution feature. We calculated the growth rate of the NJUEBMI population, the historical prevalence and incidence of ESRD to estimate future changes in ESRD patient population. The annual costs were adjusted for inflation by the health care consumer price index (CPI), and were analyzed and forecasted to evaluate the sustainable development of health care expenditures. Additionally, the annual percentage change (APC) for the forecasted variables from 2015 to 2025 was calculated using SAS 9.4.

We plotted the historical and forecasted values, providing the upper and lower 95 % confidence limits for the forecasted values. The diagram provided a visual representation of the data and the estimated range for the 10-year projection period. We also listed the goodness-of-fit statistical values from the forecasting models to examine the accuracy of the forecasted values.

## Results

### Number of the NJUEBMI Population

**Fig. 1:**
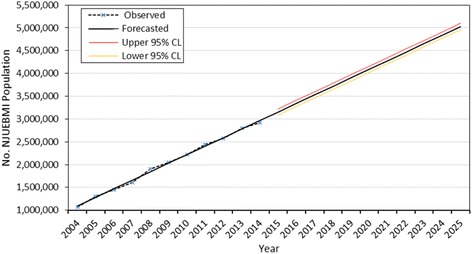
Forecasted NJUEBMI population counts according to the linear trend model. The asterisk and dashed line represent the observed data, the solid line indicates the forecasted counts, and the lines above and below the solid line from 2015 to 2025 indicate the upper and lower 95 % confidence limits (CL), respectively, for the forecasted counts

The size of the NJUEBMI population was forecasted by using the linear trend model that best fit the data and minimized the residuals. As shown in Fig. 1, the number of the NJUEBMI population steadily increased throughout 2004–2014, the asterisk and dashed line represented the actual historical data, the solid line indicated the forecasted counts, and the lines above and below the solid line from 2015 to 2025 indicated the upper and lower 95 % confidence limits, respectively, for the forecasted counts. Because all other forecasted variables appeared similar, we did not present them all. According to the forecasting model, the average annual growth rate of NJUEBMI population counts between 2015 and 2025 was forecasted to be approximately 4.77 %, with the absolute counts being forecasted as 3,156,022 in 2015 (95 % CI, 3,087,175-3,224,869), 4,092,646 in 2020 (95 % CI, 4,023,799-4,161,493), and 5,029,270 in 2025 (95 % CI, 4,960,423-5,098,117) (Table 1).

**Table 1:** Observed and forecasted values for selected years^a^

	2009	2014	2015	2020	2025	APC
						Period (2015–2025)
Number of NJUEBMI population (No.) (Linear Trend)^b^	2,032,074	2,968,697	3,156,022	4,092,646	5,029,270	4.77 %
	2,050,731	2,921,065	(3,087,175–3,224,869)	(4,023,799–4,161,493)	(4,960,423–5,098,117)	
Incidence (pmp)						
All ESRD (Linear Trend)	205.9	219.8	222.6	236.5	250.5	1.19 %
	206.8	218.8	(219.8–225.4)	(233.7–239.4)	(247.7–253.3)	
HD (Linear Trend)	171.5	183.5	185.9	197.9	209.9	1.22 %
	171.2	182.5	(184.2–187.6)	(196.2–199.6)	(208.2–211.7)	
PD (Damped Trend Exponential Smoothing)	22.0	27.9	28.9	34.3	39.8	3.25 %
	22.4	27.4	(26.9–30.9)	(32.0–36.6)	(37.2–42.3)	
Kidney Transplant (Log Linear (Holt) Exponential Smoothing)	12.4	8.6	8.1	5.8	4.1	−6.58 %
	13.2	8.9	(7.0–9.4)	(4.9–6.7)	(3.4–5.0)	
Prevalence (pmp)						
All ESRD (Linear Trend)	1083	1215	1241	1373	1505	1.95 %
	1083	1228	(1186–1297)	(1318–1428)	(1450–1560)	
HD (Damped Trend Exponential Smoothing)	815.9	928.8	967.4	1070	1128	1.55 %
	820.2	942.5	(915.4–1019)	(765.5–1374)	(529.1–1727)	
PD (Linear Trend)	74.0	120.6	129.9	176.6	223.2	5.56 %
	71.7	125.3	(124.5–135.4)	(171.1–182.0)	(217.7–228.7)	
Kidney Transplant (Linear Trend)	195.7	150.3	141.2	95.8	50.4	−9.79 %
	190.7	160.6	(128.6–153.8)	(83.2–108.4)	(37.8–63.0)	
Health care costs (¥, in millions)						
All ESRD (Linear (Holt) Exponential Smoothing)	172.0	318.9	341.5	470.9	600.3	5.80 %
	168.5	315.6	(323.9–359.0)	(427.7–514.1)	(541.8–658.9)	
HD (Linear Trend)	160.5	271.7	294.0	405.3	516.5	5.80 %
	153.5	275.5	(279.3–308.7)	(390.6–419.9)	(501.9–531.2)	
PD (Damped Trend Exponential Smoothing)	7.8	32.2	34.1	45.9	54.6	4.82 %
	10.5	31.1	(30.1–38.1)	(18.3–73.5)	(−4.0–113.2)	
Kidney Transplant (Linear Trend)	5.3	9.0	9.8	13.5	17.3	5.85 %
	4.5	9.0	(8.6–10.9)	(12.4–14.7)	(16.1–18.4)	
Per capita medical expenses (¥, in thousands)						
All ESRD (Logistic Linear Trend)	76.1	90.3	92.1	97.1	99.0	7.25‰
	75.9	87.9	(89.2–94.2)	(96.0–97.9)	(98.6–99.3)	
HD (Linear (Holt) Exponential Smoothing)	88.7	104.7	106.5	120.8	135.0	2.40 %
	91.2	100.1	(98.8–114.3)	(112.2–129.4)	(125.7–144.4)	
PD (Logistic Linear Trend)	68.2	92.3	94.4	98.9	99.8	5.58‰
	71.3	84.9	(84.9–98.1)	(96.9–99.6)	(99.4–99.9)	
Kidney Transplant (Linear Trend)	12.7	20.5	22.1	29.9	37.8	5.51 %
	11.6	19.2	(19.6–24.5)	(27.5–32.4)	(35.3–40.2)	

*Abbreviations*: *APC* annual percentage change, *NJUEBMI* Nanjing urban employee basic medical insurance, *ESRD* end stage renal disease, *HD* hemodialysis, *PD* peritoneal dialysis, *pmp* per million population

^a^The top values for each variable are the forecasted values. The bottom values for each variable in the 2009 and 2014 columns are the observed values. The bottom values in the 2015, 2020, and 2025 columns are 95 % confidence intervals for the forecasts

^b^The selected forecasting models are listed in the brackets below each variable, separately

### Incidence of ESRD

The incidence of ESRD was forecasted using linear trend (Table 1). From 2015 to 2025, the incidence of ESRD was projected to slowly increase by approximately 1.19 % annually, with forecasted values of 222.6 pmp in 2015 (95 % CI, 219.8–225.4), 236.5 pmp in 2020 (95 % CI, 233.7–239.4), and 250.5 pmp in 2025 (95 % CI, 247.7–253.3). Three subgroups of ESRD, hemodialysis (HD), peritoneal dialysis (PD) and kidney transplant, were forecasted using linear trend, damped trend exponential smoothing, and log linear (holt) exponential smoothing, respectively (Table 1). Both incidence of HD and PD increased at average annual growth rate of 1.22 and 3.25 %, respectively, whereas the incidence of kidney transplant was forecasted to decrease by 6.58 % annually.

### Prevalence of ESRD

The prevalence of ESRD was also forecasted by using linear trend (Table 1). From 2015 to 2025, the prevalence of ESRD would increase by approximately 1.95 % annually, with the predictive values of 1241 pmp in 2015 (95 % CI, 1186–1297), 1373 pmp in 2020 (95 % CI, 1318–1428), and 1505 pmp in 2025 (95 % CI, 1450–1560). The prevalence of HD, PD and kidney transplant, was forecasted by using damped trend exponential smoothing, linear trend and linear trend, respectively (Table 1). Similar to the incidence, the prevalence of HD and PD was projected to increase by 1.55 and 5.56 %, the prevalence of kidney transplant decreased by 9.79 % annually.

### Health care costs of ESRD

The health care costs of ESRD were forecasted by using log linear (Holt) exponential smoothing trend (Table 1). The ESRD health care costs were projected to increase at an average annual growth rate of approximately 5.80 %, from 2015 to 2025. The forecasted values were 341.5 million Chinese Yuan (CNY) in 2015 (95 % CI, 323.9–359.0), which was equivalent to 52.6 million USA Dollars (USD), 470.9 million CNY (72.5 million USD) in 2020 (95 % CI, 427.7–514.1), and 600.3 million CNY (92.4 million USD) in 2025 (95 % CI, 541.8–658.9). The health care costs of HD, PD and transplant, were projected to increase, using linear trend, damped trend exponential smoothing, and linear trend, respectively (Table 1).

### Per capita medical expenses of ESRD

The per capita medical expenses of ESRD were forecasted by using logistic linear trend (Table 1). From 2015 to 2025, the per capita ESRD medical expenses were expected to increase at growth rate of approximately 7.25‰ annually, with forecasted values of 92.1 thousand CNY (14.2 thousand USD) in 2015 (95 % CI, 89.2–94.2), 97.1 thousand CNY (14.9 thousand USD) in 2020 (95 % CI, 96.0–97.9), and 99.0 thousand CNY (15.2 thousand USD) in 2025 (95 % CI, 98.6–99.3). The per capita medical expenses of HD, PD and transplant, were forecasted to increase, by using linear (Holt) exponential smoothing, logistic linear trend and linear trend, respectively (Table 1). Of the three subgroups, the per capita medical expenses of kidney transplant were the lowest.

### Goodness-of-Fit statistical values

We analyzed all of the selected forecasting models for the best fit of the data, and listed the goodness-of-fit statistical values in Table 2. The mean forecasting errors for the variables ranged from −0.94 to 1.17, with mean percent errors of −2.49 to 5.62 %, reflecting the deviations from the observed data. The R-square values, ranging from 0.756 to 0.997, assumed that the models had high degree of fit and correlation between the actual and forecasted values.

**Table 2:** Goodness-of-fit statistical values

	Mean error^a^	Maximum error	Minimum error	Mean percent error^b^	Maximum percent error	Minimum percent error	R-square^c^
Number of NJUEBMI population (No.)	2.27E-05	55591	−47633	−0.09	2.93	−2.88	0.997
Incidence (pmp)							
All ESRD	3.23E-09	1.57	−1.55	−3.07E-03	0.73	−0.75	0.943
HD	−3.28E-09	0.94	−0.98	−1.73E-03	0.53	−0.54	0.971
PD	0.15	1.52	−0.65	0.56	5.66	−2.76	0.869
Kidney Transplant	−0.12	0.83	−1.04	−1.42	6.29	−10.68	0.756
Prevalence (pmp)							
All ESRD	4.55E-09	58.32	−43.33	−0.07	5.36	−4.77	0.915
HD	1.17	24.73	−65.08	0.12	2.78	−8.16	0.931
PD	−2.73E-10	4.7	−4.92	0.41	10.93	−5.61	0.993
Kidney Transplant	−3.09E-09	10.31	−9.57	−0.06	6.42	−4.9	0.961
Health care costs (¥, in millions)							
All ESRD	−0.64	16.61	−15.22	−1.57	7.09	−13.46	0.990
HD	−1.55E-09	8.89	−12.8	0.63	15.31	−12.41	0.991
PD	0.49	3.83	−1.61	5.62	37.36	−9.99	0.969
Kidney Transplant	−1.00E-10	0.95	−0.88	−0.36	29	−23.87	0.953
Per capita medical expenses (¥, in thousands)							
All ESRD	−0.11	3.35	−2.89	−0.27	3.88	−4.48	0.942
HD	0.48	4.79	−4.6	0.45	5.34	−5.38	0.777
PD	−0.94	8.46	−8.69	−2.49	9.46	−13.08	0.852
Kidney Transplant	−2.50E-11	1.94	−1.34	−0.7	10.93	−12.1	0.917

*Abbreviations*: *NJUEBMI* Nanjing urban employee basic medical insurance, *ESRD* end stage renal disease, *HD* hemodialysis, *PD* peritoneal dialysis, *pmp* per million population

^a^The mean error indicates an average difference (2004 to 2014) of the forecasted values from the observed values. The maximum error indicates one of the 11 years (2004 to 2014) that exhibited the largest deviation of the forecasted value from the observed value, and the minimum error indicates the smallest deviation. A positive sign indicates over forecasting, whereas a negative sign indicates under forecasting

^b^The mean percent error reflects a proportional deviation of the mean error. Maximum and Minimum percent errors reflect proportional deviations of the largest and smallest errors, respectively

^c^The R-Square indicates the correlation between the observed values and the forecasted values

## Discussion

For decades, the Chinese government has been committing to reforming the national health care system and providing medical insurance for its people. Of three types of basic medical insurance, UEBMI provides the highest proportion of health care reimbursement in China. In Nanjing, an increasing number of urban residents have participated in NJUEBMI during the past ten years, from 16 % of the Nanjing population in 2004, to 36 % in 2014 (Additional file 1: Table S1). The NJUEBMI population would increase steadily at an average annual growth rate of approximately 4.77 %. The ESRD patients in NJUEBMI population pay 5–10 % of the costs of RRT. Thus, RRT is affordable for NJUEBMI population, the treatment gap between needed and actual RRT could be neglected, and the data from this population might reflect the true burden of ESRD in urban residents in relatively developed areas of China.

Our study demonstrated trends of increasing ESRD incidence in NJUEBMI population. The incidence of ESRD increased to 218.8 pmp in 2014, which was higher than in Beijing [3], Shanghai [4] and Hong Kong [6], but much lower than in Taiwan [5, 6]. International comparisons by USRDS [6] and other national reports on ESRD epidemiology [8–12] showed that the incidence of ESRD in NJUEBMI population was higher than in the United Kingdom, Austria, New Zealand and Brazil; lower than in the USA, Japan, Brunei and Singapore; similar to Malaysia and Thailand. The incidence of ESRD in NJUEBMI population was forecasted to increase at an annual growth rate of 1.19 % and reach 250.5 pmp by 2025. There are several reasons for the increasing trend of ESRD incidence. First, the per capita disposable income, medical insurance coverage and reimbursement proportion are improving, thus, more patients could afford RRT. Second, the life expectancy is rising in China, with the continuous increase of the aging population, the incidence of chronic diseases is increasing, including CKD [13]. Third, the increasing incidence of metabolic diseases, such as hypertension, diabetes, dyslipidemia and obesity cause a rapid increase of CKD [14]. Other risk factors, such as unhealthy behaviors, occupational and environmental stress, iatrogenic and biotoxic factors might contribute to the increase of CKD as well [15, 16]. These factors will lead to a future increase in ESRD incidence in mainland China.

The prevalence of ESRD in NJUEBMI population had a similar trend to incidence, increased to 1228pmp in 2014, much higher than in Beijing [3] and Shanghai [4], lower than in Taiwan [5, 6], and similar to Hong Kong [6]. Compared with international reports on ESRD epidemiology [8–12], the prevalence of ESRD in NJUEBMI population was higher than in the United Kingdom, Austria, New Zealand, Brazil, Malaysia and Thailand; and lower than in the USA, Japan, Brunei and Singapore. Some epidemiological investigations have demonstrated that Asian descent was a specific high-risk ethnic group for CKD [17]. Asia was estimated to have the highest number of patients needing RRT. However, the proportion actually receiving RRT ranged from 17 to 34 %[18]. Previous reports [5, 6, 8–12] have shown that the prevalence of ESRD in developed regions of Asia (e.g., Taiwan, Japan, Singapore and Brunei) were higher than in most Western countries (i.e., the United Kingdom, Austria and New Zealand) and higher than in Latin America (e.g., Brazil). Then, we hypothesized that the incidence and prevalence of ESRD in mainland China should not be lower than in most Western countries.

The incidence and prevalence of HD and PD showed increasing trends similar to that of ESRD, but the incidence and prevalence of kidney transplant trended in an opposite direction. The prevalence of kidney transplant in NJUEBMI population decreased to 160.6 pmp in 2014, was much lower than in Austria and New Zealand [9]. Unlike in situation in Western countries, few Chinese individuals are willing to be live donors, and Chinese kidney transplantation depends on deceased donors [19], especially executed prisoners [20]. The Chinese government has required all hospitals to stop transplanting organs from executed prisoners immediately. Civilian organ donation is the sole source for organ transplantation in China [20]. For the above reasons, as in our forecasted trends, the incidence and prevalence of kidney transplant would decrease in the following decade.

With both number of NJUEBMI population and prevalence of ESRD increasing, the total health care costs for ESRD are rapidly increasing annually. In 2014, the total RRT costs were ¥ 315.6 million ($ 48.6 million), among which, a total of ¥ 270.6 million ($ 41.6 million) was paid by the NJUEBMI; this sum served 1.23‰ of NJUEBMI population, consumed approximately 2.94 % of the annual health care budget costs of the NJUEBMI [21]. In high income countries and regions, health care spending for ESRD is one of the major contributors to rising health care costs. In the USA and Taiwan, the RRT cost for ESRD patients served 1.98‰ and 2.90‰ of the general population, accounted for 5.6 and 6.0 % of the annual medicare budget, respectively, in 2012 [6, 22]. The annual increase in spending for RRT globally ranged from 6 to 12 % over the last two decades and continues to grow, particularly in low- and middle-income countries [23]. Although the health care costs of ESRD in NJUEBMI population would increase by approximately 5.80 % annually, lower than global average level, the rapid increasing health care costs of ESRD will still bring heavy burden for Nanjing government.

The per capita medical expenses for ESRD in NJUEBMI population increased steadily during the last decade and would continue to grow. Compared with previous reports [6, 24–26], our results were similar to Shanghai, and much lower than in the USA and most developing countries of sub-Saharan Africa. Except for inflation, the improvement of RRT service should be the main reason for annually increases [27]. Of three RRT subgroups, the per capita medical expenses of HD were the highest, and the expenses for kidney transplant were the lowest. Kidney transplant has the best outcomes and the highest cost-effect [28, 29]. To improve the outcomes of ESRD patients and control medical expenses, kidney transplant should be encouraged.

Although the future variables of ESRD in NJUEBMI population might not change as forecasted here, the predicted growth in the ESRD burden remains useful for policy makers and financial planners. If the forecasted trends continue, the RRT costs for ESRD will reach ¥ 600.3 million ($ 92.4 million) in NJUEBMI population in 2025, which represents a heavy financial burden on the Nanjing government. The pressures on health care provider systems will also rise, with increasing demands for nephrologists, nurses, technicians and transplant related programs.

Our study has several strengths. Previously published reports on ESRD epidemiology in mainland China were based on data from multiple dialysis facilities [3, 4, 30]. Our study was based on an entire population covered by NJUEBMI, and the ESRD patients in NJUEBMI had smallest treatment gaps between needed and actual RRT in Nanjing population. Furthermore, we obtained continuous data from 2004 to 2014 with complete registration of ESRD patients.

The study has some limitations. First, the 95 % confidence limits provided precision for the estimates, on the basis of the trends for actual data from 2004 to 2014. These limits, changing in breadth as the forecast lead-time increases, suggested that the reliability of the forecasted values was variable. Second, in time-series models, we presumed to know nothing about the causality that affected the variables we were trying to forecast, these forecasts were only based on historical data, and the trends might also be influenced by various factors. For example, the incidence of HD and PD might be changed by different dialysis initiating time, the doctors’ advice and patients’ choices; the growth rates of incidence and prevalence of ESRD might be slowed down, if more effective CKD prevention and intervention strategies are used [31]. So these forecasting models could be reliable in the short run, and the predicted data in long term could only be used for reference. Finally, our study retrospectively analyzed the data from the NJUEBMI population, which might only reflex the status of ESRD in urban residents in developed areas in China. Further studies on ESRD epidemiology should be performed in the entire Nanjing population and the whole Chinese population.

## Conclusions

The modeled projections firstly demonstrate that the incidence and prevalence of ESRD will continue to increase up to 2025 in NJUEBMI population. The total health care costs and per capita medical expenses for ESRD will also increase. These data suggest the urgency of developing and implementing effective ESRD prevention strategies.

## Abbreviations

CKD, chronic kidney disease; CNRDS, Chinese renal data system; CNY, Chinese Yuan; CPI, consumer price index; ERA-EDTA, European renal association–European dialysis and transplant association; ESRD, end stage renal disease; HD, hemodialysis; NJMIIS, Nanjing medical insurance information system; NJRCMI, Nanjing rural cooperative medical insurance; NJUEBMI, Nanjing urban employee basic medical insurance; NJURBMI, Nanjing urban resident basic medical insurance; PD, peritoneal dialysis; pmp, per million population; RCMI, rural cooperative medical insurance; RRT, renal replacement therapy; TSN, Taiwan society of nephrology; UEBMI, urban employee basic medical insurance; URBMI, urban resident basic medical insurance; USD, USA Dollar; USRDS, United States renal data system

## Additional file

Additional file 1: Table S1. The data on the development of the population, economy and social insurance in Nanjing City (2004–2014). (DOCX 14 kb)
