# Supplementary material for: Forecast of the incidence, prevalence and burden of end-stage renal disease in Nanjing, China to the Year 2025
Source: BMC Nephrol. 2016 Jun 13;17:60. doi: 10.1186/s12882-016-0269-8 (PMC4906971; doi:10.1186/s12882-016-0269-8)
Supplement: Additional file 1: Table S1. — The data on the development of the population, economy and social insurance in Nanjing City (2004–2014). (DOCX 14 kb) [file 12882_2016_269_MOESM1_ESM.docx]

Table S1. The data on the development of the population, economy and social insurance in Nanjing City (2004-2014)*.

|  | 2004 | 2005 | 2006 | 2007 | 2008 | 2009 | 2010 | 2011 | 2012 | 2013 | 2014 |
| --- | --- | --- | --- | --- | --- | --- | --- | --- | --- | --- | --- |
| Resident Population (10,000) | 668.18 | 689.8 | 719.06 | 741.3 | 758.89 | 771.31 | 800.76 | 812.00 | 816.10 | 818.78 | 821.61 |
| UEBMI Population  (%)** | 1075013  (16.1) | 1301882  (18.9) | 1447189  (20.1) | 1611073  (21.7) | 1900340  (25.0) | 2050731  (26.6) | 2225205  (27.8) | 2441197  (30.1) | 2577020  (31.6) | 2802096  (34.2) | 2921065  (35.6) |
| Per capita disposable income of urban residents (CNY)  (Growth ratio %) | 11602  (13.8) | 14997  (19.9) | 17538  (16.9) | 20317  (15.9) | 23123  (13.8) | 25504  (10.3) | 28312  (11.0) | 32200  (13.7) | 36322  (12.8) | 39881  (9.8) | 42568  (8.8) |
| Per capita disposable income of rural residents (CNY)  (Growth ratio %) | 5533  (12.4) | 6225  (12.5) | 7045  (13.2) | 8020  (13.8) | 8951  (11.6) | 9858  (10.1) | 11128  (12.9) | 13108  (17.8) | 14786  (12.8) | 16531  (11.8) | 17661  (10.3) |
| Basic medical insurance coverage (%) | NA | NA | NA | NA | NA | > 98.0 | > 98.0 | > 98.0 | 98.7 | 98.1 | > 98.0 |
| Total GDP (Billion CNY)  (Growth ratio %) | 1910  (17.3) | 2413  (15.2) | 2773.7  (15.1) | 3283.7  (15.7) | 3814.6  (12.1) | 4230.2  (11.5) | 5012.6  (13.1) | 6145.5  (12.0) | 7201.5  (11.7) | 8011.7  (11.0) | 8820.8  (10.1) |

Abbreviations: UEBMI, Urban employee basic medical insurance; CNY, Chinese Yuan; NA, not available.

*All the data were obtained from the annual report on the development of the Nanjing population by Nanjing Population and Family Planning Commission, and the annual report on the Nanjing economic and social development by Nanjing Municipal Bureau of Statistics.

**Numbers in the brackets were presented as the percentage of the resident population.
